# Supplementary material for: An Interactive Curriculum to Teach Person-Centered Contraceptive Counseling
Source: MedEdPORTAL. 2023 Dec 19;19:11368. doi: 10.15766/mep_2374-8265.11368 (PMC10728363; doi:10.15766/mep_2374-8265.11368)
Supplement: Supplementary file 1 — Contraceptive Options Chart and Pocket Guide.pdfPerson-Centered Contraceptive Counseling Module folderCase Development Tool.docxFacilitator Information and SP Training.docxFormative Session Checklist.docxPre- and Postsurveys.docx [file mep_2374-8265.11368-s001.zip › C. Case Development Tool.docx]

*MedEdPORTAL* Standardized Patient Case Development Tool

Date: June – September 2022

Primary Case Author: Irene Tang

Secondary Case Author: Devon Rupley, MD

Standardized Patient Educator: Zachary Milligan and Mark Friedlander

Name of Case: Contraceptive Counseling OSCE

Name of Educational and/or Assessment Activity: Patient-Centered Contraceptive Counseling

Patient Name: Jane Smith

Chief Complaint: “talk about birth control”

Most Likely Diagnosis and Differential with Rationale From History and/or Physical Exam: N/A

Challenge Question: N/A

Domains: Check all that apply

- Professionalism

☑ Communication and Interpersonal Skills

☑ Medical History

- Physical Exam

☑ Shared Decision-Making

☑ Patient Education

- Clinical Reasoning
- Documentation
- Handoff
- Presentation
- Other:

Type and Level of Learner: clinical-year medical student

Case Objectives: Please list specific objectives for each of the domains you have checked above:

1. Explain the mechanism of action and effectiveness of contraceptive methods.

2. Describe risks, benefits, and uses of contraceptive methods, including for emergency contraception.

3. Gather appropriate patient history to inform contraceptive options counseling.

4. Employ shared decision-making techniques while counseling a standardized patient.

5. Appreciate the importance of patient-centered counseling in a variety of clinical settings.

| SETTING: outpatient, in patient, ED, home, nursing home, rehab, group, etc. | Outpatient, telehealth setting |
| --- | --- |
| PATIENT PROFILE: Information about the “patient” that helps select an SP and helps the learner get an understanding of them as a person. SP will know more information about the patient than learner will ever ask but allows SP to portray a fully developed patient personality. If none of the items below are particulars for the case, please write “all may be used.” | |
| Age range | 20 |
| Religious/spiritual background | Not specified |
| Sex (e.g., male, female, intersex, transwoman, transman) | Female |
| Sexual orientation (e.g., heterosexual, lesbian, gay, bisexual, pansexual, queer, asexual) | Bisexual |
| Gender expression (e.g., man, woman, genderqueer) | Woman |
| Race and ethnicity | Not specified |
| Physical description (e.g., BMI, height range) | BMI 26 kg/m^2^ |
| Physical limitations | None |
| Patient appearance (e.g., disheveled, hospital gown, business casual, casual) | Casual |
| Moulage + location (e.g., none, bruises, scars, body piercing, tattoos) | None |
| Affect (e.g., pleasant, cooperative) | Neutral to positive, not embarrassed about answering any questions about her sexual history |
| Family group (e.g., who is family, who they live with) | Lives in college dorm |
| Education | Current college sophomore |
| Level of health literacy | High |
| Employment, if any - present and past, noting any current stresses | None |
| Home/homeless - type of dwelling, number of stories, owned or rented | Lives in college dorm |
| Financial situation - any current stresses | None |
| Insurance status (e.g., un/under/insured, public/private, HMO/PPO) | Insured |
| Habits (i.e., diet, exercise, caffeine, smoking, alcohol, drugs) | Uses alcohol socially and cannabis occasionally |
| Activities (i.e., hobbies, sports, clubs, friends) | Not specified |
| Typical day - what is the usual daily routine | Not specified |

| CASE INFORMATION | |
| --- | --- |
| Chief Concern: What the patient will say when greeted by the student. The patient’s primary reason for seeking medical care often stated in their own words. | “I want to talk about options for birth control.” |
| Additional Concerns: Other, if any, concerns the patient has today (i.e., symptoms, requests, expectations, etc.) that will become part of set agenda. | N/A |
| THE PATIENT’S STORY: The SP will be asked to tell their symptom story and the personal and emotion impact for each of their concerns. You will want to write this in the patient’s voice. The symptom story should be able to answer this question: “Tell me more about [chief concern/additional concern], starting at the beginning and bringing me up to now.”  The personal context should be able to answer questions concerning the broader personal/psychosocial context of symptoms, especially the patient’s beliefs/attributions.  The emotional context should be able to ask how are you doing with this, how does this make you feel, how has this affected you emotionally? IMPACT: How has this affected your life? How has this been for your family? | “I’ve only used condoms in the past. I like them, but I’m wondering if I should use something else as well. I’m a little concerned about the risk of failure or the condom breaking.”  “I do not want to get pregnant for many years. I’m looking for a birth control method that is easy to use and more effective than condoms. I know people that use the pill or the shot, and I’ve heard of a bunch of other methods but don’t know that much about them. I’m looking for more information in general.”  “I would be fine with the diaphragm if it’s more effective. I’m fine with something that has hormones. I’m fine with taking pills, but I’m not sure I can take a pill consistently at the same time every day. I’m interested in hearing more about the weekly patch or the monthly ring. I don’t love the idea of getting a shot every 3 months. I like the idea of not having to take a pill every day, and I’m fine with having something placed in my arm or uterus. I would be happy with my periods becoming lighter or going away and have no problem with some irregularity. I’m not interested in something that could make my periods heavier or more painful.”  “I’m interested in learning how this method works and how effective it is. Also, I’m worried that this method may make me gain weight because it happened to one of my friends.” |
| HISTORY OF PRESENT ILLNESS: Although some of the HPI will be given in the patient’s symptom story, the learners will expand the story during the direct question section. Below, describe the detailed history, usually about the chief concern, which the student must develop in order to make a useful assessment of the problem: | |
| Onset (when; gradual or sudden) | N/A |
| Setting (what was going on or where was patient when symptoms first noticed?) | N/A |
| Duration (how long) | N/A |
| Time relationships (frequency, constant or intermittent) | N/A |
| Location | N/A |
| Radiation | N/A |
| Quality | N/A |
| Amount | N/A |
| Aggravated by what | N/A |
| Relieved by what | N/A |
| Associated with what | N/A |
| Attitude (what does the patient think is the problem, and how do they feel about it) | N/A |
| Overall course | N/A |
| REVIEW OF SYSTEMS: Significant positives and negatives | |
| Has migraines 1-2 times per month that last 4 hours, associated with flashing lights and tingling, relieved by Advil and rest.  All other review of systems negative. | |
| Past medical history | No diagnosed medical conditions. |
| Medication allergies (name and reaction) | None |
| Environmental allergies (name and reaction) | None |
| Illnesses | None |
| Vaccinations | Up to date |
| Surgeries | None |
| Accidents/injuries/trauma | None |
| Hospitalization | None |
|  | |
| Inclusive sexual and reproductive history | |
| Sexual practices  Sexual partners  Protection: Use of safer sex practices  Use of birth control if appropriate  Risk of intimate partner violence | Additional contraceptive history: uses a condom every time now. Once, 6 months ago, had an encounter without a condom but was so anxious afterwards that now she always wants to use one. Is confident asking partners to use condoms. One of her partners has occasionally asked not to use a condom, but she has always insisted. Plans to use condoms in the future unless she is with an exclusive partner because she knows they can protect from STIs.  Sexual history: identifies as bisexual. Not currently in a relationship. Has had two regular male partners over the past 3 months. Has vaginal, oral, and anal intercourse. Is satisfied with her sex life and has no concerns. Has never been pressured to have sex, never felt unsafe, and never felt she had to have sex when she didn’t want to. Had one prior partner a few years ago (identifies as nonbinary, assigned female at birth, uses they/them pronouns). |
| OB/GYN history | Age of onset of menses: 13  Age of menopause: N/A  Number of pregnancies: 0  Number of live births: 0  Number of miscarriages: 0  Number of abortions: 0  First day of last period: two weeks ago  Period symptoms: pretty heavy and painful, often late or early by a few days, last about 5 days. On day 2, soaks through a regular tampon and changes her tampon every 3-4 hours. Sometimes has had to miss school due to pain.  HPV vaccine: had during childhood, was on schedule with all vaccines  Pap smear: none (per guidelines, start at age 21)  Prior STIs: never |
| Medications | Prescription/dose/reason: none  Over the counter/dose/reason: Advil as needed for cramps and headaches  Herbs/supplements/dose/reason: none  Other: none |
| Immunizations | ☑ Tetanus  ☑ Flu  ☑ Hepatitis   - Pneumovax – N/A   ☑ HPV  ☑ Other – all childhood vaccinations on schedule |
| Tobacco products:   - Cigarettes - Cigar - Pipe - Chew - E-cigarettes | ☑ Never   - Past - year started/year quit - Current   - Quantity   - # of years |
| Alcohol  ☑ Beer  ☑ Wine  ☑ Liquor   - Other | - Never - Past - year started/year quit   ☑ Current   - - Quantity – on average 4 drinks on weekends   - # of years – not specified |
| Drugs  ☑ Weed   - Cocaine - Heroin - Meth - IV - Inhalants - Other | - Never - Past - year started/year quit   ☑ Current   - - Quantity – smokes cannabis 2 times a month   - # of years – not specified |
| Diet (describe) | Not specified |
| Exercise (describe) | Not specified |
| List any other important social history or information important to this case | None |
| Family history |  |
| Mother, father, siblings, grandparents, and other significant findings | Maternal grandmother – stroke, around age 70, still alive  Paternal grandmother – breast cancer, diagnosed around age 60, still alive  Parents, younger sister healthy |
|  |  |
| Physical Exam - List exam maneuvers expected for this case and any abnormal findings that SP will simulate. (tenderness, hyper-hypo reflex, rebound, weakness, etc.)  N/A | |
| PHYSICAL EXAM FINDINGS |  |
| 1. Written in layperson’s terms | N/A |
| 1. General appearance - affect, appearance, position of patient at opening (i.e., sitting, lying down, holding abdomen, etc.) | N/A |
| 1. Vital signs | T 37 HR 72 BP 110/70 RR 12 O2 100% on RA |
| 1. Specific findings and affect | N/A |
| 1. Response to certain physical movements | N/A |
|  |  |
| DIAGNOSIS AND DIFFERENTIAL |  |
| Diagnosis with support from positive and negative history and PE findings | N/A |
| Differential with support from positive and negative history and PE findings | N/A |
|  |  |
| MANAGEMENT OR DIAGNOSTIC PLAN | The student should recognize that combined hormonal contraceptive methods are contraindicated due to the history of migraine with aura. The student should suggest contraceptive methods in line with the patient’s goals and articulate a plan for next steps. |
|  |  |
| PROFESSIONALISM ISSUES OR CHALLENGES | The student should use a shared decision-making approach to provide patient-centered, non-directive counseling and educate the patient about different contraceptive options. |
