# Supplementary material for: An Interactive Curriculum to Teach Person-Centered Contraceptive Counseling
Source: MedEdPORTAL. 2023 Dec 19;19:11368. doi: 10.15766/mep_2374-8265.11368 (PMC10728363; doi:10.15766/mep_2374-8265.11368)
Supplement: Supplementary file 1 — Contraceptive Options Chart and Pocket Guide.pdfPerson-Centered Contraceptive Counseling Module folderCase Development Tool.docxFacilitator Information and SP Training.docxFormative Session Checklist.docxPre- and Postsurveys.docx [file mep_2374-8265.11368-s001.zip › D. Facilitator Information and SP Training.docx]

**Facilitator Information and Standardized Patient Training**

This document was provided to preceptors and standardized patients to prepare them for the formative session. It contains information about the logistics of holding the session as well as the case description and script for standardized patients to follow during the session.

Objectives

This session covers the following Clerkship Learning Objectives:

- Acquire knowledge of routine OB/Gyn care from reproductive years through menopause, and of normal and abnormal reproductive function.
- Reinforce skills for taking a reproductive health history and performing a trauma-informed physical and pelvic examination.
- Hone patient communication skills.

In addition, this session covers the following Entrustable Professional Activity (EPA) and critical competencies as described in AAMC’s Core Entrustable Professional Activities for Entering Residency Curriculum Developers’ Guide. Domains of competence include Patient Care (PC); Knowledge for Practice (KP); Interpersonal and Communication Skills (ICS); and Professionalism (P).

- EPA 1: Gather a history and perform a physical examination
- PC 2: Gather essential and accurate information about patients and their conditions through history-taking, physical examination, and the use of laboratory data, imaging, and other tests
- PC 5: Make informed decisions about diagnostic and therapeutic interventions based on patient information and preferences, up-to-date scientific evidence, and clinical judgment
- PC 7: Counsel and educate patients and their families to empower them to participate in their care and enable shared decision making
- KP 1: Demonstrate an investigatory and analytic approach to clinical situations
- ICS 1: Communicate effectively with patients, families, and the public, as appropriate, across a broad range of socioeconomic and cultural backgrounds
- ICS 7: Demonstrate insight and understanding about emotions and human responses to emotions that allow one to develop and manage interpersonal interactions
- P 1: Demonstrate compassion, integrity, and respect for others
- P 3: Demonstrate respect for patient privacy and autonomy
- P 5: Demonstrate sensitivity and responsiveness to a diverse patient population, including but not limited to diversity in gender, age, culture, race, religion, disabilities, and sexual orientation

Logistics
Total session time: 9:00 am - 12:45 pm

- Sessions conducted on Zoom
- 3 preceptors (senior students, residents/fellows, and/or faculty) needed for each session
- Standardized patients (SPs) and preceptors log on 30 minutes before first student encounter to go over logistics and any case/checklist questions before students arrive
- One 15-minute break/buffer time in the middle of the sessions

Timing per encounter (30 minutes total):

- 2-3 minutes for student orientation/door note/Zoom troubleshooting
- 15 minutes for the encounter, with 5-minute warning
- 10 minutes for feedback
- 2-3 minute buffer/break

Schedule template:

| Time (SPs called at 9:00) | Student | SP | Preceptor |
| --- | --- | --- | --- |
| 9:30 (Encounter 1) | Student 1 | SP 1 | Preceptor 1 |
|  | Student 2 | SP 2 | Preceptor 2 |
|  | Student 3 | SP 3 | Preceptor 3 |
|  |  |  |  |
| 10:00 (Encounter 2) | Student 4 | SP 1 | Preceptor 1 |
|  | Student 5 | SP 2 | Preceptor 2 |
|  | Student 6 | SP 3 | Preceptor 3 |
|  |  |  |  |
| 10:30 (Encounter 3) | Student 7 | SP 1 | Preceptor 1 |
|  | Student 8 | SP 2 | Preceptor 2 |
|  | Student 9 | SP 3 | Preceptor 3 |
|  |  |  |  |
| 11:00 – 11:15 | Break/buffer | | |
|  |  |  |  |
| 11:15 (Encounter 4) | Student 10 | SP 1 | Preceptor 1 |
|  | Student 11 | SP 2 | Preceptor 2 |
|  | Student 12 | SP 3 | Preceptor 3 |
|  |  |  |  |
| 11:45 (Encounter 5) | Student 13 | SP 1 | Preceptor 1 |
|  | Student 14 | SP 2 | Preceptor 2 |
|  | Student 15 | SP 3 | Preceptor 3 |
|  |  |  |  |
| 12:15 (Encounter 6) | Student 16 | SP 1 | Preceptor 1 |
|  | Student 17 | SP 2 | Preceptor 2 |
|  | Student 18 | SP 3 | Preceptor 3 |

Information for students

About one week before the session, students are sent an email with the schedule, door note, outline of the debrief checklist, and link to the online module to prepare for the session.

Door note

This is a telehealth encounter. You will have 15 minutes in this encounter, followed by about 10 minutes for debriefing. You may leave the Zoom meeting when you are finished with the debriefing.

You are rotating at the undergraduate student health clinic. Your preceptor, Dr. O, asks you to speak with the next patient first.

CC: “talk about birth control”

Name: Jane Smith

Pronouns: she/her

Age: 20

Vitals: T 37 HR 72 BP 110/70 RR 12 O_2_ 100% on RA

BMI: 26 kg/m^2^

Dr. O asks you to collect the patient history as relevant to contraceptive options, explore the patient’s preferences for contraceptive methods, provide the patient with appropriate information to help her make an informed decision, and begin to arrive at a shared decision with the patient. The plan is for Dr. O to join you in continuing to counsel the patient after this encounter.

Script for standardized patient

*Note: encounter will probably be more fluid than typical OSCEs; sections may not be in a particular order.*

*Italics denote extra information to say if specifically asked.*

Case summary: Jane Smith is a 20-year-old college sophomore who is coming to the student health clinic to learn more about her birth control options.

Character affect: Neutral to positive, not embarrassed about answering any questions about her sexual history.

History of present illness/chief concern: No health complaints. She wants to talk about options for birth control.

Contraceptive history:

- Prior methods: only condoms
- Satisfaction with method: somewhat, but wondering if she should use something else as well
  - Why? A little concerned about risk of failure/condom breaking. Asks if student can tell her how much the risk is.
- Condom use self-efficacy:
  - How often do you use a condom? Every time now. *Once, 6 months ago, had an encounter without a condom but was so anxious afterwards that now she always wants to use one*
  - Confident asking partner to use condom? Yes
  - Ever felt pressured not to use a condom? One of her partners has occasionally asked not to use a condom, but she has always insisted.
  - Plans to use condoms in the future? She knows they can protect from STIs, so she is planning to use them unless she is with an exclusive partner.

Sexual history:

- Orientation: identifies as bisexual
- Relationship status: not currently
- Recent sexual activity: two regular male partners (over the past 3 months)
  - Types of intercourse—vaginal, oral, anal? All
- Satisfied with sex life? Yes, *would not change anything about her sex life, has no concerns, never feels bad/not enjoyable for her*
- Ever experienced reproductive coercion? No, *has never been pressured to have sex, never felt unsafe, never felt she had to have sex when she didn’t want to*
- Prior partners: just one a few years ago, *identifies as nonbinary, assigned female at birth, uses they/them pronouns*
- Knowledge about STI risk: no questions, uses condoms to protect against STIs

Gynecologic history:

- First period (“menarche”): age 13
- First day of last period: two weeks ago
- Period symptoms: pretty heavy and painful, *often late or early by a few days,*
- *last about 5 days*
  - How many pads/tampons on heaviest day(s)? On day 2, changes tampon every 3-4 hours, *soaks through a regular tampon*
  - Ever had to miss work/school due to pain? Sometimes
- HPV vaccine: had during childhood, was on schedule with all vaccines
- Pap smear: none (per guidelines, start at age 21)
- Prior STIs: never
- Prior pregnancies: none

Past medical history: “Not really”

- Headaches/migraines? Yes, migraines
  - Tell me more? 1-2x per month, last about 4 hours
  - Associated symptoms? Flashing lights and tingling as it comes on
  - Take anything for them/anything help? Advil and rest
- Any other conditions (student may ask about blood clots, hypertension, ischemic heart disease, etc.)? No

Medications: “Not really”

- Over the counter? Advil sometimes
  - What for? Cramps and headaches
  - Headaches? See above

Allergies: None

Surgical history: None

Family history:

- Maternal grandmother – stroke, *around age 70, still alive*
- Paternal grandmother – breast cancer, *diagnosed around age 60, still alive*
- Parents, younger sister healthy

Social history:

- Alcohol? Socially, *on average 4 drinks on weekends*
- Cannabis? Occasional, *smokes 2x a month*
- Smoking or vaping tobacco, using other substances? No

Contraceptive counseling:

- Reproductive goals/thoughts on pregnancy? Does not want to get pregnant for a while, like many years.
- What are you looking for in a birth control method? Something easy to use and better than condoms (*more effective*)
- What birth control options have you been considering/have you heard about?
  - She knows people that use the pill or the shot. She’s heard of a bunch of other methods but doesn’t know that much about them.
  - She’s looking for more information in general.
  - She’s interested in learning about other methods if the student asks.
- How do you feel about…
  - (Student may describe various methods or features. Ideally students will identify that migraine with aura is a contraindication to combination pills/patch/ring, and barrier methods are less effective.)
  - Barrier methods (diaphragm): fine; does it work better than condoms?
  - Pills (without specifying need for consistency): fine
  - Pill that needs to be taken at same time every day: not sure she can be that consistent
  - Hormones: fine
  - Weekly patch, monthly ring: interested in hearing more
  - Shot every 3 months: doesn’t love the idea
  - Something that stays in the body for years/might need a doctor’s visit to discontinue/IUD/implant (“LARC”): likes idea of not having to take a pill every day, fine with placement in arm or uterus
  - Periods becoming lighter or irregular or going away: interested, no problem with it
  - Periods becoming heavier/more painful: not interested
- What questions do you have? (Questions to ask if not answered already)
  - How well does [method] work? (Opportunity for students to describe stats)
  - How does [method] work to prevent pregnancy?
  - Does [method] lead to weight gain? A friend had that side effect with [the pill/shot], or she heard about that happening with [other method].
- Summary/what are your thoughts?
  - Not sure…patient lists out several aspects of the different methods described that she likes or dislikes
  - Would like to think about the options
  - Student may offer to schedule follow-up, provide reading materials, etc.

Debrief

SP will share screen and provide feedback as they fill out the checklist.
