# Supplementary material for: An Interactive Curriculum to Teach Person-Centered Contraceptive Counseling
Source: MedEdPORTAL. 2023 Dec 19;19:11368. doi: 10.15766/mep_2374-8265.11368 (PMC10728363; doi:10.15766/mep_2374-8265.11368)
Supplement: Supplementary file 1 — Contraceptive Options Chart and Pocket Guide.pdfPerson-Centered Contraceptive Counseling Module folderCase Development Tool.docxFacilitator Information and SP Training.docxFormative Session Checklist.docxPre- and Postsurveys.docx [file mep_2374-8265.11368-s001.zip › E. Formative Session Checklist.docx]

**Formative Session Checklist**

This checklist was used to assess student performance after the standardized patient session. It is intended to take less than 10 minutes to complete.

Person-Centered Contraceptive Counseling Scale

From the perspective of the patient, think about your visit. How do you think your provider did? Please rate them on each of the following.

|  | Poor | Fair | Good | Very good | Excellent |
| --- | --- | --- | --- | --- | --- |
| Respecting me as a person |  |  |  |  |  |
| Letting me say what mattered to me about my birth control method |  |  |  |  |  |
| Taking my preferences about my birth control seriously |  |  |  |  |  |
| Giving me enough information to make the best decision about my birth control method |  |  |  |  |  |

Scale adapted from:

1. Dehlendorf C, Fox E, Silverstein IA, et al. Development of the Person-Centered Contraceptive Counseling scale (PCCC), a short form of the Interpersonal Quality of Family Planning care scale. *Contraception*. 2021;103(5):310-315. doi:10.1016/j.contraception.2021.01.008

Medical History-Taking

Did the student assess the presence of the following contraindications to combined hormonal methods?

|  | Done | Not done |
| --- | --- | --- |
| Cigarette smoking |  |  |
| Migraine with aura |  |  |
| History of blood clots (DVT/PE) |  |  |
| BONUS: Hypertension |  |  |
| BONUS: Ischemic heart disease |  |  |
| BONUS: Current breast cancer |  |  |
| BONUS: less than 6 weeks postpartum (*e.g.*, assessed whether patient had any previous pregnancies) |  |  |

Did the student obtain the following gynecologic history?

|  | Done | Not done |
| --- | --- | --- |
| Asked when last menstrual period began |  |  |
| Asked about patient's typical menstrual cycles (*e.g.*, regularity, heaviness, pain) |  |  |
| BONUS: Assessed satisfaction with current menstrual cycles |  |  |

Did the student obtain the following contraceptive history?

|  | Done | Not done |
| --- | --- | --- |
| Asked about prior use of any methods |  |  |
| BONUS: Asked about satisfaction with prior methods |  |  |

Did the student obtain the following sexual history?

|  | Done | Not done |
| --- | --- | --- |
| Assessed partners and practices |  |  |
| Assessed consistency of barrier method use |  |  |
| Asked about history of STIs |  |  |
| BONUS: Assessed knowledge about STI risk |  |  |
| BONUS: Assessed condom use self-efficacy |  |  |
| BONUS: Assessed satisfaction with sex life |  |  |
| BONUS: Screened for reproductive coercion |  |  |

Counseling Skills

Did the student perform the following counseling skills?

|  | Done | Not done |
| --- | --- | --- |
| Mirrored language |  |  |
| Communicated without medical jargon |  |  |
| Approached sensitive subject matters without judgment |  |  |
| Identified patient's reproductive goals (*e.g.*, desire for and timing of future pregnancy) |  |  |
| Explored patient’s preferences for features of contraceptive methods (*e.g.*, desire for/tolerance of amenorrhea; acceptance of hormones) |  |  |
| Communicated method effectiveness and/or pregnancy risk clearly |  |  |
| Suggested additional methods in line with patient’s values |  |  |
| Assessed patient’s confidence and ability to use method correctly and consistently (*e.g.*, explored potential barriers to use) |  |  |
| Checked understanding |  |  |
| Addressed concerns and/or misconceptions respectfully |  |  |
| Summarized plan for next steps in line with patient's goals |  |  |

Open-Ended Questions

One thing done well:

________________________________________________________________

________________________________________________________________

________________________________________________________________

________________________________________________________________

________________________________________________________________

One thing that could be improved:

________________________________________________________________

________________________________________________________________

________________________________________________________________

________________________________________________________________

________________________________________________________________

Other comments:

________________________________________________________________

________________________________________________________________

________________________________________________________________

________________________________________________________________

________________________________________________________________

Email to share results:

________________________________________________________________
