# Supplementary material for: An Interactive Curriculum to Teach Person-Centered Contraceptive Counseling
Source: MedEdPORTAL. 2023 Dec 19;19:11368. doi: 10.15766/mep_2374-8265.11368 (PMC10728363; doi:10.15766/mep_2374-8265.11368)
Supplement: Supplementary file 1 — Contraceptive Options Chart and Pocket Guide.pdfPerson-Centered Contraceptive Counseling Module folderCase Development Tool.docxFacilitator Information and SP Training.docxFormative Session Checklist.docxPre- and Postsurveys.docx [file mep_2374-8265.11368-s001.zip › F. Pre- and Postsurveys.docx]

**Pre- and Post-Surveys**

These 5-10 minute surveys were used to evaluate the effectiveness of the new contraceptive counseling curriculum by assessing contraceptive knowledge, skills, and attitudes before and after the curriculum took place.

Pre-survey:

Knowledge (set A; participants randomized to be shown set A or B)

The following questions relate to your knowledge of contraceptive methods, side effects, and contraindications.

1. A medical visit is required for insertion and removal for which of the following methods?
   1. Copper IUD
   2. Levonorgestrel IUDs
   3. Diaphragm
   4. a & b
   5. All of the above
2. Levonorgestrel IUDs work by:
   1. Thickening the cervical mucus
   2. Thinning out the uterine lining
   3. Levonorgestrel is toxic to sperm
   4. a & b
3. Potential side effects of Medroxyprogesterone (DMPA), brand name Depo-Provera, include:
   1. Menstrual irregularities
   2. Weight gain
   3. Decreased bone density
   4. Delay in return to fertility
   5. All of the above
4. Which of the following methods is most effective?
   1. Condoms (internal or external)
   2. Nexplanon implant
   3. Combined hormonal contraceptives (pills, patch, or ring)
   4. Depo-Provera
5. Which of the following is a common side-effect of the hormonal implant (e.g. Nexplanon)?
   1. Weight gain
   2. Intermittent and unpredictable bleeding
   3. Reduced efficacy of antibiotics
   4. All of the above
   5. None of the above
6. A 30-year-old never smoker with a history of migraine with aura is interested in starting Yaz (combined hormonal oral contraceptive pill). What is your concern?
   1. She may experience more severe or frequent migraines
   2. This could interact with her sumatriptan, which she uses as a migraine abortive agent
   3. Starting combined hormonal contraception increases the risk of stroke in patients with migraines with aura
   4. Starting ANY form of hormonal contraception increases the risk of stroke in patients with migraines with aura
   5. No concerns
7. Women with well-controlled hypertension may be able to use which of the following contraceptive methods?
   1. Combined hormonal contraceptives (pills, patch, or ring)
   2. Progestin-only methods (pills or depot injections)
   3. Intrauterine device or system
   4. Emergency contraception
   5. All of the above
   6. None of the above
8. Which of the following forms of contraception may be used to improve menorrhagia?
   1. Combined hormonal contraceptives (pills, patch, or ring)
   2. Progestin-only methods (pills or depot injections)
   3. Mirena IUD
   4. All of the above
   5. None of the above
9. A young woman who is a patient of yours has a history of pelvic inflammatory disease (PID). She continues to have unprotected intercourse with multiple partners. This patient should NOT have an IUD placed because it will further increase her risk for PID. This statement is:
   1. True
   2. False
10. Which of the following is a potential benefit of combined oral contraceptives?
    1. Decreased risk of ovarian cancer
    2. Improvement of acne
    3. Reduced menstrual cramps
    4. All of the above

Correct answers: 1. D, 2. D, 3. E, 4. B, 5. B, 6. C, 7. E, 8. D, 9. B, 10. D

Knowledge (set B; participants randomized to be shown set A or B)

The following questions relate to your knowledge of contraceptive methods, side effects, and contraindications.

1. A medical visit is required for insertion and removal for which of the following methods?
   1. Implant
   2. Diaphragm
   3. Nuvaring
   4. a & b
   5. b & c
   6. All of the above
2. What is the efficacy rate of the Copper IUD (Paragard)?
   1. 75%
   2. 85%
   3. 99%
   4. 100%
3. Potential side effects of Medroxyprogesterone (DMPA), brand name Depo-Provera, include:
   1. Migraine
   2. Increase in size of ovarian cysts
   3. Increased risk of blood clots
   4. Amenorrhea
   5. All of the above
4. Which of the following methods is most effective?
   1. Spermicide
   2. Mirena IUD
   3. Progestin-only pill
   4. Fertility awareness
5. Which of the following is a potential risk of Nexplanon insertion?
   1. Infection
   2. Bruising at the insertion site
   3. Need for removal by a healthcare provider
   4. All of the above
6. A 45-year-old obese patient with hypertension is interested in starting a combined hormonal oral contraceptive pill. What is your concern?
   1. She may experience weight gain
   2. She may experience unpredictable menstrual cycles
   3. Starting combined hormonal contraceptives in women aged 35 and older increases risk of thromboembolism
   4. Starting combined hormonal contraceptives may increase her risk of endometrial cancer
   5. No concerns
7. True or false: The pill (combined hormonal contraceptives) and the ring can be used in extended cycles.
   1. True
   2. False
8. Which of the following forms of contraception may be used to improve menorrhagia?
   1. ParaGard IUD
   2. Hormonal implant (Nexplanon)
   3. Spermicide
   4. All of the above
   5. None of the above
9. Which of the following can be used for emergency contraception?
   1. Paragard IUD
   2. Mirena IUD
   3. Oral progestin
   4. All of the above
10. What is the mechanism of action of the copper IUD?
    1. Thickening the cervical mucus
    2. Inhibiting ovulation
    3. Causing toxicity to sperm
    4. a & b

Correct answers: 1. A, 2. C, 3. D, 4. B, 5. D, 6. C, 7. A, 8. B, 9. D, 10. C

Knowledge questions were adapted from:

1. Worthington RO, Oyler J, Pincavage A, Baker NA, Saathoff M, Rusiecki J. A novel contraceptive counseling and shared decision-making curriculum for internal medicine residents. *MedEdPORTAL*. Published online December 4, 2020:11046. doi:10.15766/mep_2374-8265.11046
2. Innovating Education in Reproductive Health. Contraception 101. Published May 8, 2019. Accessed March 18, 2022. https://www.innovating-education.org/2019/05/contraception-101/

Career plans

1. Which of the following career plans are you considering? (select all that apply)
   1. Ob-gyn
   2. Other primary care specialty (pediatrics, internal medicine, family medicine)
   3. Non-primary care specialty
   4. Other ________________________________________________
   5. No idea

Skills and attitudes

The following questions relate to your interests and attitudes regarding contraceptive counseling.

1. How important to you is learning about contraceptive counseling during medical school?
   1. Not at all
   2. Slightly
   3. Moderately
   4. Very
   5. Extremely
2. How relevant is learning about patient-centered counseling to your career goals?
   1. Not at all
   2. Slightly
   3. Moderately
   4. Very
   5. Extremely
3. Please indicate your comfort level with the following (response options: 1 = I need close supervision from a preceptor; 2 = I need distant or indirect supervision from a preceptor; 3 = I can perform independently in some situations; 4 = I can perform independently in most situations; 5 = I can teach this skill to others)
   1. Initiating a conversation about the patient’s reproductive goals
   2. Reviewing contraception options including the option of no contraception
   3. Discussing contraception side effects
   4. Discussing contraception efficacy (i.e. failure rates)
   5. Exploring the patient’s perceptions and beliefs surrounding various contraception options
   6. Negotiate a decision for contraception in partnership with the patient

Additional comments

1. We welcome any additional comments you may have.

________________________________________________________________

Post-survey:

The following sections were shown in the pre-survey:

- Knowledge (randomized to set A or set B)
- Career plans
- Skills and attitudes
- Additional comments

The following sections were shown in the post-survey:

- Knowledge (set A or set B, whichever was not shown in the pre-survey)
- Pocket guide use
- Skills and attitudes
- Additional comments

Knowledge (set A; participants shown the set they were not shown on the pre-survey)

The following questions relate to your knowledge of contraceptive methods, side effects, and contraindications.

1. A medical visit is required for insertion and removal for which of the following methods?
   1. Copper IUD
   2. Levonorgestrel IUDs
   3. Diaphragm
   4. a & b
   5. All of the above
2. Levonorgestrel IUDs work by:
   1. Thickening the cervical mucus
   2. Thinning out the uterine lining
   3. Levonorgestrel is toxic to sperm
   4. a & b
3. Potential side effects of Medroxyprogesterone (DMPA), brand name Depo-Provera, include:
   1. Menstrual irregularities
   2. Weight gain
   3. Decreased bone density
   4. Delay in return to fertility
   5. All of the above
4. Which of the following methods is most effective?
   1. Condoms (internal or external)
   2. Nexplanon implant
   3. Combined hormonal contraceptives (pills, patch, or ring)
   4. Depo-Provera
5. Which of the following is a common side-effect of the hormonal implant (e.g. Nexplanon)?
   1. Weight gain
   2. Intermittent and unpredictable bleeding
   3. Reduced efficacy of antibiotics
   4. All of the above
   5. None of the above
6. A 30-year-old never smoker with a history of migraine with aura is interested in starting Yaz (combined hormonal oral contraceptive pill). What is your concern?
   1. She may experience more severe or frequent migraines
   2. This could interact with her sumatriptan, which she uses as a migraine abortive agent
   3. Starting combined hormonal contraception increases the risk of stroke in patients with migraines with aura
   4. Starting ANY form of hormonal contraception increases the risk of stroke in patients with migraines with aura
   5. No concerns
7. Women with well-controlled hypertension may be able to use which of the following contraceptive methods?
   1. Combined hormonal contraceptives (pills, patch, or ring)
   2. Progestin-only methods (pills or depot injections)
   3. Intrauterine device or system
   4. Emergency contraception
   5. All of the above
   6. None of the above
8. Which of the following forms of contraception may be used to improve menorrhagia?
   1. Combined hormonal contraceptives (pills, patch, or ring)
   2. Progestin-only methods (pills or depot injections)
   3. Mirena IUD
   4. All of the above
   5. None of the above
9. A young woman who is a patient of yours has a history of pelvic inflammatory disease (PID). She continues to have unprotected intercourse with multiple partners. This patient should NOT have an IUD placed because it will further increase her risk for PID. This statement is:
   1. True
   2. False
10. Which of the following is a potential benefit of combined oral contraceptives?
    1. Decreased risk of ovarian cancer
    2. Improvement of acne
    3. Reduced menstrual cramps
    4. All of the above

Correct answers: 1. D, 2. D, 3. E, 4. B, 5. B, 6. C, 7. E, 8. D, 9. B, 10. D

Knowledge (set B; participants shown the set they were not shown on the pre-survey)

The following questions relate to your knowledge of contraceptive methods, side effects, and contraindications.

1. A medical visit is required for insertion and removal for which of the following methods?
   1. Implant
   2. Diaphragm
   3. Nuvaring
   4. a & b
   5. b & c
   6. All of the above
2. What is the efficacy rate of the Copper IUD (Paragard)?
   1. 75%
   2. 85%
   3. 99%
   4. 100%
3. Potential side effects of Medroxyprogesterone (DMPA), brand name Depo-Provera, include:
   1. Migraine
   2. Increase in size of ovarian cysts
   3. Increased risk of blood clots
   4. Amenorrhea
   5. All of the above
4. Which of the following methods is most effective?
   1. Spermicide
   2. Mirena IUD
   3. Progestin-only pill
   4. Fertility awareness
5. Which of the following is a potential risk of Nexplanon insertion?
   1. Infection
   2. Bruising at the insertion site
   3. Need for removal by a healthcare provider
   4. All of the above
6. A 45-year-old obese patient with hypertension is interested in starting a combined hormonal oral contraceptive pill. What is your concern?
   1. She may experience weight gain
   2. She may experience unpredictable menstrual cycles
   3. Starting combined hormonal contraceptives in women aged 35 and older increases risk of thromboembolism
   4. Starting combined hormonal contraceptives may increase her risk of endometrial cancer
   5. No concerns
7. True or false: The pill (combined hormonal contraceptives) and the ring can be used in extended cycles.
   1. True
   2. False
8. Which of the following forms of contraception may be used to improve menorrhagia?
   1. ParaGard IUD
   2. Hormonal implant (Nexplanon)
   3. Spermicide
   4. All of the above
   5. None of the above
9. Which of the following can be used for emergency contraception?
   1. Paragard IUD
   2. Mirena IUD
   3. Oral progestin
   4. All of the above
10. What is the mechanism of action of the copper IUD?
    1. Thickening the cervical mucus
    2. Inhibiting ovulation
    3. Causing toxicity to sperm
    4. a & b

Correct answers: 1. A, 2. C, 3. D, 4. B, 5. D, 6. C, 7. A, 8. B, 9. D, 10. C

Pocket guide use

1. Have you used the contraceptive pocket guide in any of the following settings? (select all that apply)
   1. During patient encounter in ob-gyn clerkship
   2. During patient encounter in another rotation (please specify) ________________________________________________
   3. During contraceptive counseling practice session (if applicable)
   4. None

Skills and attitudes

The following questions relate to your interests and attitudes regarding contraceptive counseling.

1. How important to you is learning about contraceptive counseling during medical school?
   1. Not at all
   2. Slightly
   3. Moderately
   4. Very
   5. Extremely
2. How relevant is learning about patient-centered counseling to your career goals?
   1. Not at all
   2. Slightly
   3. Moderately
   4. Very
   5. Extremely
3. Please indicate your comfort level with the following (response options: 1 = I need close supervision from a preceptor; 2 = I need distant or indirect supervision from a preceptor; 3 = I can perform independently in some situations; 4 = I can perform independently in most situations; 5 = I can teach this skill to others)
   1. Initiating a conversation about the patient’s reproductive goals
   2. Reviewing contraception options including the option of no contraception
   3. Discussing contraception side effects
   4. Discussing contraception efficacy (i.e. failure rates)
   5. Exploring the patient’s perceptions and beliefs surrounding various contraception options
   6. Negotiate a decision for contraception in partnership with the patient

Additional comments

1. We welcome any additional comments you may have.

________________________________________________________________
