# Supplementary material for: An Interactive Curriculum to Teach Person-Centered Contraceptive Counseling
Source: MedEdPORTAL. 2023 Dec 19;19:11368. doi: 10.15766/mep_2374-8265.11368 (PMC10728363; doi:10.15766/mep_2374-8265.11368)
Supplement: Supplementary file 1 — Contraceptive Options Chart and Pocket Guide.pdfPerson-Centered Contraceptive Counseling Module folderCase Development Tool.docxFacilitator Information and SP Training.docxFormative Session Checklist.docxPre- and Postsurveys.docx [file mep_2374-8265.11368-s001.zip › _Educational Summary Report_11368.pdf]

MedEdPORTAL® The AAMC Journal of Teaching and Learning Resources

Original Publication Open Access

An Interactive Curriculum to Teach Person-Centered Contraceptive Counseling

Irene Tang, MD*, Devon Rupley, MD

*Corresponding author: irenedt@uw.edu

Abstract

Introduction: Following the *Dobbs v. Jackson Women's Health Organization* Supreme Court decision, it is increasingly important for all providers to be equipped to counsel on contraceptive options. Current curricula are insufficient for medical students to attain competency in contraceptive counseling. Quality contraceptive counseling requires patient-centered communication skills, which are also critical in many other clinical scenarios. Systematic teaching of patient-centered communication is lacking, both in contraceptive counseling and more broadly. **Methods:** We developed a person-centered contraceptive counseling curriculum containing a reference guide, 5- to 10-minute interactive online module, and 30-minute formative standardized patient session for clerkship-year medical students. Performance during formative sessions was evaluated using a checklist, with standardized patients and preceptors providing real-time feedback. We used surveys of knowledge, self-perceived skills, and attitudes about patient-centered counseling to compare students who did and did not receive the curriculum. **Results:** Twenty-seven students received the new curriculum. The reference guide and online module were easily integrated into a clinical rotation without requiring additional time spent by educators. The formative session required more resources to implement but was valuable for students to solidify the communication skills in the new curriculum. Checklist results showed that students demonstrated many of the counseling skills taught in the module. Survey results about the impact of the new curriculum were promising but limited by the small sample size. **Discussion:** The curriculum successfully introduced patient-centered contraceptive counseling skills and provided a valuable practice opportunity. Other sites could adapt components of this curriculum to enhance education in person-centered contraceptive counseling.

Keywords
Contraception, Patient-Centered Communication, Shared Decision-Making, Communication Skills, OB/GYN, Primary Care, Standardized Patient, Women's Health

Educational Objectives

By the end of this activity, learners will be able to:

1. Gather appropriate patient history to inform contraceptive options counseling.
2. Elicit reproductive goals and preferences to inform contraceptive recommendations.
3. Employ patient-centered language to share information about contraceptive methods (e.g., mechanism of action, effectiveness, risks, benefits, and/or uses).
4. Utilize a shared decision-making tool to aid in counseling and patient education.

Citation:
Tang I, Rupley D. An interactive curriculum to teach person-centered contraceptive counseling. *MedEdPORTAL*. 2023;19:11368.
https://doi.org/10.15766/mep_2374-8265.11368

Introduction

Following the *Dobbs v. Jackson Women's Health Organization* Supreme Court decision in June 2022,[1] which has resulted in significant reduction in access to abortion services as well as a shortage of OB/GYN providers across the country, it has become increasingly important for all health care providers to be equipped to counsel patients on contraceptive options.[2] Although national organizations recommend incorporating contraceptive management into routine primary care visits, rates have been poor overall and lower in visits with generalists, pediatricians, and other specialists compared to obstetrician-gynecologists.[3] One critical barrier to delivering contraceptive care is inadequate physician education. A study of fourth-year medical students from schools across the United States revealed that standard medical school curricula provided insufficient training for students to attain competency in contraceptive counseling, a core learning objective for medical students

Copyright © 2023 Tang and Rupley. This is an open-access publication distributed under the terms of the Creative Commons Attribution license.

outlined by the Association of Professors of Gynecology and Obstetrics.[4] Similarly, researchers have found gaps in contraceptive knowledge and discomfort with counseling among internal medicine residents.[5]

Contraceptive counseling is unique and challenging for several reasons. There can be multiple medically appropriate contraceptive options for a patient, and patients have different preferences for and prior knowledge about contraceptive methods.[6] In addition, there is a history of contraceptive coercion in the United States; biases have been shown to affect contraceptive counseling, such that patients from more marginalized groups have experienced more pressure to choose highly effective contraceptive methods.[6,7] Because of these factors, as well as the personal nature of the topic, providing contraceptive counseling in a patient-centered manner is preferred and has been shown to improve patient satisfaction and contraceptive use.[8]

A patient-centered model that is well suited to contraceptive counseling is shared decision-making. Shared decision-making lies between two extremes—directive counseling, a paternalistic approach where the provider aims to promote certain methods, and informed choice, where patient autonomy is prioritized and the provider only shares information about methods.[8] In practice, shared decision-making in contraceptive counseling has been described as an iterative process of eliciting patient preferences, sharing information, and helping the patient arrive at a decision.[6] Beyond the context of contraceptive counseling, patient-centered communication and shared decision-making are growing areas of interest throughout medical education.[9,10] These approaches are applicable in a variety of settings, but systematic teaching of the required skills is lacking in medical schools, especially during clinical rotations.[11]

In a literature search, our team was unable to locate existing curricula on person-centered contraceptive counseling for medical students. A curriculum designed for medical residents was notable for its dual focus on contraceptive knowledge and shared decision-making technique; however, it lacked opportunities for learners to practice their communication skills and receive feedback, including input from patients.[5] Building upon this educational approach, we designed a multimodal curriculum to teach a shared decision-making framework for contraceptive counseling. Our curriculum included a visual aid, both to serve as a reference for students and because decision support tools have been shown to facilitate patient-centered communication about contraceptive options.[8] We also designed a short interactive module that used an analogy to illustrate a patient-centered communication framework; analogies can be effective tools for learning complex ideas by mapping them to familiar concepts.[12] Lastly, standardized patient encounters are a well-documented method to provide opportunities for students to practice skills in a low-stakes environment, so we developed a standardized patient case as part of our person-centered contraceptive counseling curriculum.[13]

Methods

In 2022, we developed a multimodal curriculum at Columbia University's medical school to teach students to provide patient-centered contraceptive counseling. We integrated the new curriculum into the OB/GYN clerkship for two rotation blocks, with a total of 27 third-year students. The new curriculum was provided in addition to the preexisting clerkship curriculum, which we considered the baseline curriculum. The baseline curriculum contained only a 1-hour lecture on contraception, which was generally delivered by a family planning fellow using a premade slide deck over Zoom. The lecture focused on risks, benefits, and mechanisms of action for the different contraceptive methods. While it included one slide on counseling that mentioned using a client-centered approach, it provided minimal actionable guidance about how to do so and did not mention shared decision-making or the use of decision support tools.

For assessment, we conducted pre- and postsurveys to evaluate the effectiveness of including the new curriculum compared to providing the baseline curriculum alone. We provided feedback on student performance during a formative session component of the new curriculum. Our project was determined to be exempt by the Columbia University Institutional Review Board.

Multimodal Curriculum

The new curriculum consisted of a contraception information pocket guide, interactive module, and formative session. Building on a previously published and widely available patient education chart developed by the University of California, San Francisco,[14] we created an expanded pocket guide to reinforce key information about contraceptive methods in a visual format that could also serve as a tool during patient encounters (Appendix A). It also included reminders about best practices for patient-centered counseling.[6,8] We distributed the pocket guide to students in hard copy and digital formats at the beginning of the OB/GYN clerkship and encouraged them to use the guide throughout the clerkship and in the formative session.

We created an interactive online module (Appendix B) designed to take 5-10 minutes to complete using the Articulate Rise 360 platform (Articulate Global). The module used an illustrated

Copyright © 2023 Tang and Rupley. This is an open-access publication distributed under the terms of the Creative Commons Attribution license.

analogy of building a tent to demonstrate a framework for person-centered contraceptive counseling, including example phrasing that students could emulate. It synthesized multiple resources about shared decision-making and contraceptive counseling methods, cited within the module. The module also included high-yield reminders for students regarding important clinical history to obtain during a contraceptive visit. We assigned the module for students to complete as preparation for the formative session during protected didactic time. We did not use any method to ensure that students had completed the module before attending the standardized patient session.

The formative session was a simulated telehealth encounter with a standardized patient followed by a feedback debriefing conversation (Appendix C). To run the session, the total staffing requirement was three preceptors, three standardized patients, and one simulation coordinator. In this implementation of the curriculum, the preceptors were an OB/GYN faculty member and two fourth-year medical students. The student preceptors met the minimum qualifications of having completed the core clinical rotations and at least one additional rotation in which they had the opportunity to practice contraceptive counseling. OB/GYN residents and fellows, as well as faculty and trainees in other specialties, could all be appropriate preceptors for this activity, as long as they have experience with and interest in patient-centered contraceptive counseling. For the simulation coordinator role, the minimum qualification was the ability to manage a Zoom meeting with breakout rooms for multiple students to participate simultaneously. The total time commitment for session facilitators was up to 4 hours for a clerkship block containing up to 18 students.

We wrote the standardized patient case with input from simulation center staff and adapted it from previously published cases.[13,15] Students were instructed to collect a targeted medical history and counsel a patient about contraceptive options using a person-centered approach and were observed one-on-one by preceptors. The session occurred on Zoom during the fourth week of the 5-week clerkship. Appendix D, which detailed the case and provided logistical information, was given to all preceptors, coordinators, and standardized patients as preparation for the session. We also recorded a video going over the case information as an optional additional training resource (the video is not included as an appendix here because it covers the same information as Appendix D). In addition, standardized patients and preceptors met for 30 minutes in the morning prior to the session to provide any necessary clarifications about the case and to ensure familiarity and uniformity regarding the session.

For the session, each student was allotted 30 minutes to complete the single 15-minute encounter, with the remainder of the time utilized for feedback. The session was formative in order to create a low-stakes environment for students to be able to practice utilizing the person-centered approach. During the debrief, the preceptor first invited the student to provide a self-reflection. Then, the standardized patient completed a checklist containing the Person-Centered Contraceptive Counseling measure,[7] which reflected patient experiences of contraceptive counseling, as well as components of a medical history relevant to contraceptive care and specific counseling skills (Appendix E). The checklist was designed to contain a comprehensive list of the important aspects of medical history to assess during an encounter for contraception; because we felt some items were more advanced than expected for a clerkship-year medical student, we denoted them as bonus items. Checklist responses were discussed in real time with the students and emailed to them afterwards. The remaining debrief time was used to provide open-ended feedback.

Assessment

As described above, we provided learners with an emailed copy of their performance based on the session checklist to use for improvement. In terms of curricular assessment, we conducted pre- and postsurveys at predetermined times to measure the impact of the new curriculum (Appendix F). Survey responses were anonymous, with a random identification number used to link each participant's responses on the two surveys. Both surveys assessed contraceptive knowledge, self-perceived confidence in counseling skills, and attitudes related to patient-centered counseling. We created two sets of knowledge questions by adapting existing contraceptive knowledge assessments.[5,16] Each student was randomized to see one of the sets on the presurvey and the other on the postsurvey. The surveys also collected information about specialty interest and use of the pocket guide.

We surveyed students from two rotation blocks receiving the baseline curriculum and two blocks receiving the additional curriculum. Several weeks after students received the existing contraception lecture, we recruited participants by sharing a QR code linking to the presurvey to assess knowledge after the standard didactic session without the influence of immediate recall. After the presurvey, the baseline group received no further contraception-specific didactics, and the new curriculum group received the online module and formative session described

Copyright © 2023 Tang and Rupley. This is an open-access publication distributed under the terms of the Creative Commons Attribution license.

above. Six weeks after completing the presurvey, we sent students an automatic email with a link to the postsurvey to assess long-term knowledge retention and changes in skills and attitudes following the remainder of the clerkship, along with the additional curriculum for those who had received it. Students were given a $10 gift card as compensation upon completing the postsurvey.

Data Analysis

We conducted statistical analyses using R version 4.0.3 (R Foundation). For survey data, we calculated composite knowledge scores by determining the number of questions answered correctly out of 10, composite skills and attitudes scores based on the means on the corresponding 5-point scales, and difference scores by subtracting prescores from postscores. We compared participants who completed both the pre- and postsurveys by curriculum received and whether they reported potential interest in a primary care specialty. We compared means using two-sample *t* tests and considered $p < .05$ statistically significant.

For checklist data, we computed mean scores on the Person-Centered Contraceptive Counseling scale and mean number of items completed in each section. We counted the number of students who completed each item of the checklist. We also examined open-ended responses.

Results

Educator Experience

A total of 27 students received the new curriculum and participated in the formative session across two clerkship rotations. Implementation of the new curriculum fit well into the flow of the current OB/GYN clerkship at our institution. The delivery of the pocket guide on the first day of the clerkship added no time and required only printing out the double-sided sheet. We asked the educator delivering the contraceptive counseling lecture to remind students of this reference sheet during the lecture and again reminded students to utilize it during the formative session. The addition of the interactive, web-based module required no educator time once it had been developed. Students reported that it was time efficient and high yield, especially as preparation for their summative standardized patient assessment.

The implementation of the formative standardized patient session required significantly more resources in terms of funding and time but was extremely valuable from an educator perspective because it gave students the opportunity to practice both contraceptive counseling and applying person-centered language in a low-stakes environment. During the initial rollout, more time was required to onboard the standardized patients, but this became less as the team grew more familiar with the case. The visit was conducted via Zoom, which gave students an opportunity to practice delivering care through telehealth. Note that this format could be utilized to record the encounter in case adequate clinical precepting is not available. Furthermore, given the focus on patient-centered counseling and the detailed checklist covering the medically necessary components of the history, the presence of clinical preceptors may not be necessary to ensure students meet the intended learning objectives, especially as the standardized patients become more familiar with the case.

Session Checklists

Table 1 shows results from the feedback checklists. On average, students scored well on the Person-Centered Contraceptive Counseling measures but were relatively weaker at providing information to help the standardized patient decide on a method. Students generally completed most of the counseling items but few history items. Less than half obtained the positive history of migraine with aura. Although many explored the patient's typical menstrual cycles and satisfaction with them, few asked when the last menstrual period began. Less than half asked several core sexual history questions, and very few completed some sexual history items identified as higher-level expectations and listed as bonus items. Many students implemented the specific counseling skills listed, including some unique to contraceptive counseling among other types of counseling.

Open-ended comments revealed several common strengths and areas for improvement. Many comments noted that students demonstrated patient-centered and respectful communication. Standardized patients wrote that they found the pocket guide useful and that it worked well in the virtual format with screensharing. Constructive feedback included several comments that students provided misinformation or did not acquire enough or any of the medical, gynecologic, or sexual history. There were also comments about students sharing too much information at once, which was often consistent with students' self-reflections during the feedback discussions.

Surveys

Fifty-six students were eligible for the surveys assessing the curriculum. Forty-one (73%) responded to the presurvey, and 27 (66%) of those completed the postsurvey (Table 2 shows curricular and participant characteristics). Among students who completed both surveys, mean scores in knowledge, skills,

Copyright © 2023 Tang and Rupley. This is an open-access publication distributed under the terms of the Creative Commons Attribution license.

Table 1. Participants' Mean Scores on Each Checklist Section and Frequency of Completion of Selected Checklist Items ($N = 27$)

Component	Results: *M* (*SD*)	Results: Total Possible: No.	Results: Done: No. (% of 27)
Section			
Person-Centered Contraceptive Counseling measure	4.4 (0.4)	5	
Respecting me as a person	4.5 (0.5)	5	
Letting me say what mattered to me about my birth control method	4.6 (0.6)	5	
Taking my preferences about my birth control seriously	4.6 (0.6)	5	
Giving me enough information to make the best decision about my birth control method	4.1 (0.6)	5	
History	8.6 (3.8)	19	
Contraindications	3.0 (1.8)	7	
Gynecologic history	1.8 (0.8)	3	
Contraceptive history	1.8 (0.5)	2	
Sexual history	2.1 (1.6)	7	
Counseling	9.7 (1.6)	11	
Item			
Contraindications: Did the student assess the presence of the following contraindications to combined hormonal methods?			
Migraine with aura			12 (44)
History of blood clots			18 (67)
Gynecologic history: Did the student obtain the following gynecologic history?			
Asked when last menstrual period began			5 (18)
Asked about patient's typical menstrual cycles			23 (85)
BONUS: Assessed satisfaction with current menstrual cycles			21 (78)
Sexual history: Did the student obtain the following sexual history?			
Assessed partners and practices			10 (37)
Assessed consistency of barrier method use			10 (37)
Asked about history of sexually transmitted infections			11 (41)
BONUS: Assessed satisfaction with sex life			2 (7)
BONUS: Screened for reproductive coercion			3 (11)
Counseling: Did the student perform the following counseling skills?			
Identified patient's reproductive goals			21 (78)
Explored patient's preferences for features of contraceptive methods			25 (93)
Suggested additional methods in line with patient's values			27 (100)
Explored potential barriers to use			22 (82)

and attitudes for the curricular groups are shown in Table 3. In the groups, different proportions of students reported being interested in OB/GYN or another primary care specialty. Of note, there was a statistically significant difference ($p = .02$) in presurvey knowledge scores between students who reported being interested in primary care ($M = 8.7$, $SD = 1.0$) and those who did not ($M = 7.6$, $SD = 1.2$). Overall, the results suggest a greater increase in knowledge scores from pre- to postsurvey in the new curriculum group (Figure); however, the difference was not significant. Self-reported confidence in skills increased from pre- to postsurvey to a similar degree in the two groups, with responses averaging between 3 (*I can perform independently in some situations*) and 4 (*I can perform independently in most situations*), as rated on a 5-point scale (1 = *I need close supervision from a preceptor*, 5 = *I can teach this skill to others*). Attitudes about the importance and relevance of learning about patient-centered counseling were positive overall and remained similar from pre- to postsurvey, with responses averaging

Table 2. Curricular and Participant Characteristics ($N = 27$)

Characteristic	Baseline Curriculum	Additional Curriculum
Description	• Preclinical: 1-hour contraceptive lecture<br>• OB/GYN clerkship: 5-week rotation with 1-hour contraceptive lecture	Baseline curriculum with additions to the OB/GYN clerkship:<br>• Pocket guide<br>• 5-minute asynchronous online module<br>• 30-minute formative standardized patient session
No. (% of 27 participants)	15 (56)	12 (44)
No. of students interested in primary care (% of students receiving curriculum)	12 (80)	5 (42)

Copyright © 2023 Tang and Rupley. This is an open-access publication distributed under the terms of the Creative Commons Attribution license.

Table 3. Participants' Pre- and Postsurvey Composite and Difference Scores Grouped by Curriculum ($N = 27$)

	M (*SD*)		
**Characteristic**	**Baseline Curriculum**	**Additional Curriculum**	***p***
Knowledge[a]			
Pre	8.5 (1.3)	8.1 (1.0)	
Post	8.5 (0.7)	8.8 (1.5)	
Change	0.0 (1.7)	0.8 (1.9)	.30
Skills[b]			
Pre	3.3 (0.7)	3.2 (0.5)	
Post	3.7 (0.6)	3.8 (0.6)	
Change	0.5 (0.6)	0.6 (0.4)	.56
Attitudes[c]			
Pre	4.9 (0.2)	4.4 (0.6)	
Post	4.9 (0.3)	4.4 (0.6)	
Change	−0.0 (0.3)	0.0 (0.4)	.80

[a]Rated out of 10.
[b]Rated on a 5-point scale (1 = *I need close supervision from a preceptor*, 5 = *I can teach this skill to others*).
[c]Rated on a 5-point scale (1 = *not at all important/relevant*, 5 = *extremely important/relevant*).

between 4 and 5, as rated on a 5-point Likert scale (1 = *not at all important/relevant*, 5 = *extremely important/relevant*).

Most of the students who received the new curriculum reported using the pocket guide in the formative session ($n = 10$, 83%). One student (8%) reported using the pocket guide in a patient encounter during the OB/GYN rotation.

Discussion

A feasible addition to the OB/GYN clerkship at our institution, our innovative curriculum provided substantial educational value while adding minimal didactic time to the rotation. Notably, although we implemented the new curriculum during the final two rotations of the core clerkship year, some students commented that the standardized patient session was the first opportunity they had had to practice contraceptive counseling in their clinical rotations. Since students have varying experiences throughout clinical rotations, it is vital to provide standardized learning experiences that ensure all students are prepared to counsel patients on contraceptive options after completing their core clerkships. An added benefit of our new curriculum is the specific opportunity to practice and receive feedback on person-centered communication skills. This kind of interactive teaching of communication skills is lacking in many medical schools during the clinical years and continues to be a gap during residency training.[11]

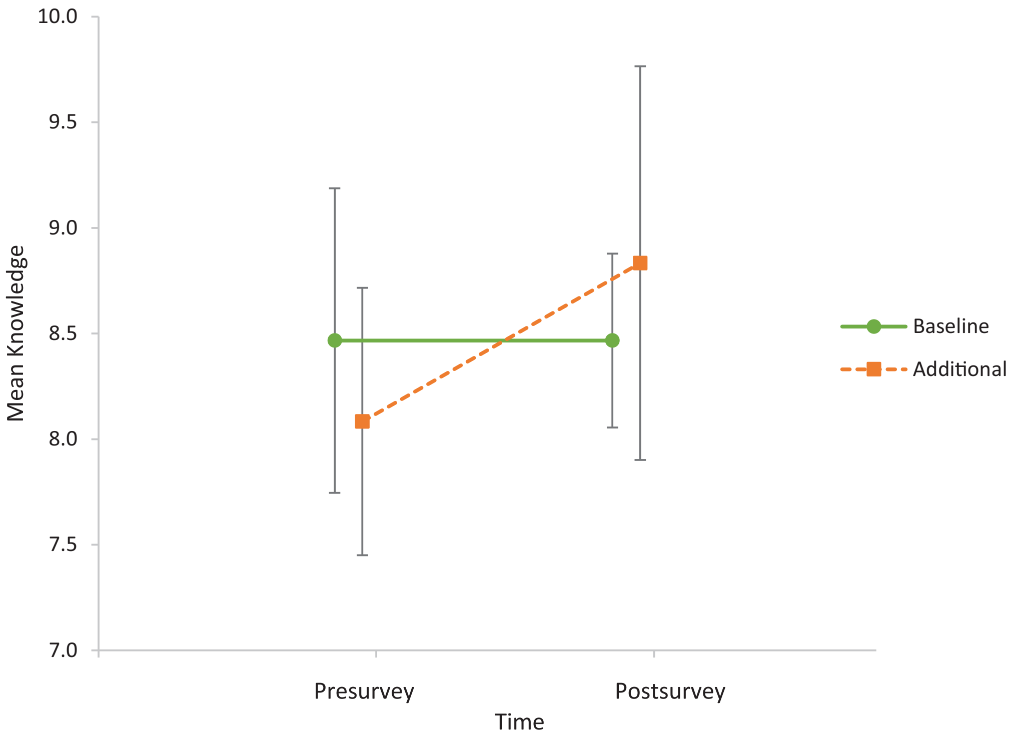

Figure. Pre- and postsurvey knowledge scores out of 10 by curriculum received among 27 medical students at Columbia University in 2022. Error bars represent 95% confidence intervals. The baseline curriculum consisted of a 1-hour contraceptive lecture in the preclinical curriculum and a 1-hour contraceptive lecture during the OB/GYN clerkship, with a total OB/GYN clerkship length of 5 weeks. The additional curriculum included a pocket guide, a 5- to 10-minute interactive online module, and a 30-minute formative standardized patient session during the OB/GYN clerkship.

Copyright © 2023 Tang and Rupley. This is an open-access publication distributed under the terms of the Creative Commons Attribution license.

Our prospective pre- and postsurvey design allowed us to assess long-term outcomes related to the new curriculum. Although our results showed that knowledge change over time was greater in the new curriculum group compared to baseline, this difference was not statistically significant, likely owing to the small sample size and potential confounding by the different proportions of students interested in primary care in the two groups. We found that the baseline and new curriculum groups experienced similar trends in self-reported confidence in skills, but the self-reflective nature of the responses limits our interpretation of this result. Future work could evaluate skills more objectively, such as by measuring performance in subsequent encounters where students must demonstrate person-centered counseling or related communication skills. The positive attitudes reported on the surveys suggest that additional contraceptive counseling education would be valued by students.

Student performance in the formative session reflected quick adaptation to a person-centered contraceptive counseling framework. Students did especially well on counseling skills emphasized in the online module, such as suggesting additional methods in line with the patient's values, indicating that the module was able to deliver specific teaching points in a short amount of time. We identified several topics that were frequently missed, such as assessing important history items and encouraging continued barrier method use, which could be better highlighted in future iterations of the interactive module. Although shared decision-making is a broad and complex skill, students were able to digest the specific framework illustrated in the online module and apply it in practice. As patient-centered communication becomes more emphasized throughout medical education, similar short and specific curricular modules can help bolster students' skills in various contexts.

Overall, the additional curriculum was well received by students and highly valued by the educational team. Students reported that "the module was time-efficient and helpful," and multiple students noted that the session was the first time they had counseled anyone on contraception. As preceptors, we appreciated the session's format because it allowed us to immediately address contraceptive knowledge gaps and offer suggestions for counseling techniques tailored to each student. Evaluation and improvement of this curriculum should be continued over a longer time frame, while also examininig the curriculum in the context of other rotations where students may be exposed to related topics. The educational objectives of this curriculum could be mapped to broader communication-related objectives that apply across medical specialties, in line with current trends of making medical education more integrated and less siloed.[17] Collecting additional qualitative feedback from students about this curriculum and evaluating the components individually would also help to identify specific areas of improvement for the curriculum design. So far, our discussions with students and observations while implementing this curriculum have revealed that students need more practice throughout medical school on information sharing and sexual history taking. Although our curriculum covered these areas, the primary focus was on person-centered counseling skills, which was reflected in student performance—the other topics would benefit from their own dedicated curricular developments.

Patient-centered contraceptive counseling is a complex task requiring generalizable communication skills and specific medical knowledge. Our multimodal curriculum targeted both areas, and the various components could be replicated separately or together at other institutions, depending on their educational needs. In formative session, our students performed well on contraceptive counseling skills, suggesting that we were successful in adapting a resident curriculum[5] and published contraceptive counseling frameworks[6,8] into educational materials appropriate for medical students. Students struggled with collecting pertinent medical histories for contraceptive care, revealing an area where further teaching and opportunities to practice are necessary. We have now permanently integrated the additional curriculum into the OB/GYN clerkship at our institution, and it will undergo continued evaluation as part of the standard clerkship curriculum moving forward. However, we purposefully designed this curriculum to be appropriate in a variety of training settings, from preclinical to clinical education, to ensure that all medical students can receive it regardless of intended specialty. Especially in the current political context, where abortion access has been markedly limited across the country, it is vital for physicians in all fields to be comfortable providing person-centered family planning care to empower patients and promote reproductive justice.[2,6,7] We encourage other medical schools to incorporate similar curricula to enhance training in both contraceptive counseling and patient-centered communication nationwide.

Appendices

A. Contraceptive Options Chart and Pocket Guide.pdf

B. Person-Centered Contraceptive Counseling Module folder

C. Case Development Tool.docx

D. Facilitator Information and SP Training.docx

Copyright © 2023 Tang and Rupley. This is an open-access publication distributed under the terms of the Creative Commons Attribution license.

E. Formative Session Checklist.docx

F. Pre- and Postsurveys.docx

All appendices are peer reviewed as integral parts of the Original Publication.

Irene Tang, MD: First-Year Resident, Department of Obstetrics and Gynecology, University of Washington School of Medicine; ORCID: https://orcid.org/0000-0002-7440-3721

Devon Rupley, MD: Assistant Professor, Department of Obstetrics and Gynecology, Columbia University Irving Medical Center

Acknowledgments
We wish to thank Aubrie Sein and Marina Catallozzi for their guidance in designing this study; Jimmy Duong for statistical support; Mark Friedlander, Zachary Milligan, Catherine Jennings, and the standardized patients who made the formative sessions possible; and the medical students who participated in this pilot curriculum with enthusiasm.

Disclosures
None to report.

Funding/Support
Dr. Irene Tang received funding from the Columbia University Scholarly Projects Program and the Sara and Arnold P. Friedman Award.

Ethical Approval
The Columbia University Institutional Review Board deemed further review of this project not necessary.

References

1. *Dobbs v Jackson Women's Health Organization*, 142 S Ct 2228 (2022).
2. Goldstein RL, Carlson JL, Tyson NA. Dobbs v. Jackson decision: it's time to get uncomfortable. *Hosp Pediatr*. 2022;12(10): e364-e366. https://doi.org/10.1542/hpeds.2022-006829
3. Bello JK, Rao G, Stulberg DB. Trends in contraceptive and preconception care in United States ambulatory practices. *Fam Med*. 2015;47(4):264-271.
4. Veazey K, Nieuwoudt C, Gavito C, Tocce K. Student perceptions of reproductive health education in US medical schools: a qualitative analysis of students taking family planning electives. *Med Educ Online*. 2015;20(1):28973. https://doi.org/10.3402/meo.v20.28973
5. Ortiz Worthington R, Oyler J, Pincavage A, Abou Baker N, Saathoff M, Rusiecki J. A novel contraception counseling and shared decision-making curriculum for internal medicine residents. *MedEdPORTAL*. 2020;16:11046. https://doi.org/10.15766/mep_2374-8265.11046
6. Chen M, Lindley A, Kimport K, Dehlendorf C. An in-depth analysis of the use of shared decision making in contraceptive counseling. *Contraception*. 2019;99(3):187-191. https://doi.org/10.1016/j.contraception.2018.11.009
7. Dehlendorf C, Fox E, Silverstein IA, et al. Development of the Person-Centered Contraceptive Counseling scale (PCCC), a short form of the Interpersonal Quality of Family Planning care scale. *Contraception*. 2021;103(5):310-315. https://doi.org/10.1016/j.contraception.2021.01.008
8. Dehlendorf C, Krajewski C, Borrero S. Contraceptive counseling: best practices to ensure quality communication and enable effective contraceptive use. *Clin Obstet Gynecol*. 2014;57(4): 659-673. https://doi.org/10.1097/GRF.0000000000000059
9. Rusiecki J, Schell J, Rothenberger S, Merriam S, McNeil M, Spagnoletti C. An innovative shared decision-making curriculum for internal medicine residents: findings from the University of Pittsburgh Medical Center. *Acad Med*. 2018;93(6):937-942. https://doi.org/10.1097/ACM.0000000000001967
10. Mitchell TO, Goldenberg MN. When doctor means teacher: an interactive workshop on patient-centered education. *MedEdPORTAL*. 2020;16:11053. https://doi.org/10.15766/mep_2374-8265.11053
11. Levinson W, Lesser CS, Epstein RM. Developing physician communication skills for patient-centered care. *Health Aff (Millwood)*. 2010;29(7):1310-1318. https://doi.org/10.1377/hlthaff.2009.0450
12. Duit R. On the role of analogies and metaphors in learning science. *Sci Educ*. 1991;75(6):649-672. https://doi.org/10.1002/sce.3730750606
13. Casey PM, Goepfert AR, Espey EL, et al. To the point: reviews in medical education—the objective structured clinical examination. *Am J Obstet Gynecol*. 2009;200(1):25-34. https://doi.org/10.1016/j.ajog.2008.09.878
14. Educational materials. Beyond the Pill. Accessed November 6, 2023. https://beyondthepill.ucsf.edu/educational-materials
15. Lupi CS, Mechaber AJ. Objective structured clinical examination: contraceptive counseling for long acting reversible methods. *MedEdPORTAL*. 2011;7:9021. https://doi.org/10.15766/mep_2374-8265.9021
16. Ramirez-McLaughlin M. Contraception 101 [video]. Innovating Education in Reproductive Health. April 9, 2019. Accessed November 6, 2023. https://www.innovating-education.org/2019/05/contraception-101/
17. Al-Eyd G, Achike F, Agarwal M, et al. Curriculum mapping as a tool to facilitate curriculum development: a new School of Medicine experience. *BMC Med Educ*. 2018;18:185. https://doi.org/10.1186/s12909-018-1289-9

Received: May 25, 2023
Accepted: September 22, 2023
Published: December 19, 2023

Copyright © 2023 Tang and Rupley. This is an open-access publication distributed under the terms of the Creative Commons Attribution license.
