# Supplementary material for: An Interactive Curriculum to Teach Person-Centered Contraceptive Counseling
Source: MedEdPORTAL. 2023 Dec 19;19:11368. doi: 10.15766/mep_2374-8265.11368 (PMC10728363; doi:10.15766/mep_2374-8265.11368)
Supplement: Supplementary file 1 — Contraceptive Options Chart and Pocket Guide.pdfPerson-Centered Contraceptive Counseling Module folderCase Development Tool.docxFacilitator Information and SP Training.docxFormative Session Checklist.docxPre- and Postsurveys.docx [file mep_2374-8265.11368-s001.zip › B. Person-Centered Contraceptive Counseling Module.zip!/B. Person-Centered Contraceptive Counseling Module/B. Person-Centered Contraceptive Counseling Module/scormcontent/assets/QaWN8sFGzKTi0one_HiekI_0aGtPfPKSX-Pocket guide.pdf]

HOW WELL DOES BIRTH CONTROL WORK?

What is your chance of getting pregnant?

★★★★★ Really, really well

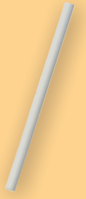

The Implant (Nexplanon)

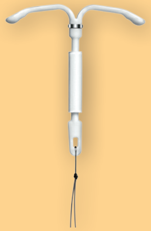

IUD (Skyla)

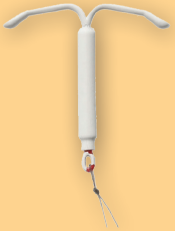

IUD (Mirena)

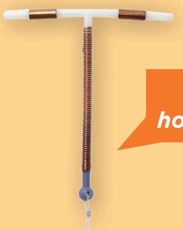

IUD (ParaGard)

No hormones

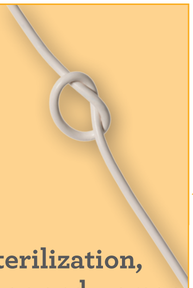

Sterilization, for men and women

Works, hassle-free, for up to...	3 years	3 years	5 years	12 years	Forever

Less than 1 in 100 women

★★★ O.K.

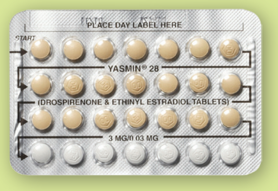

The Pill

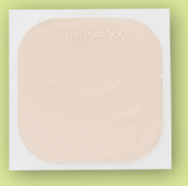

The Patch

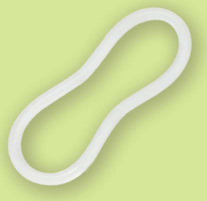

The Ring

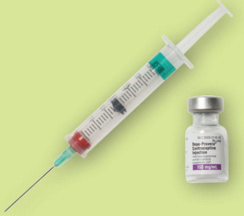

The Shot (Depo-Provera)

For it to work best, use it...	Every. Single. Day.	Every week	Every month	Every 3 months

6-9 in 100 women, depending on method

★ Not as well

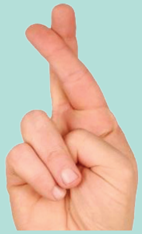

Pulling Out

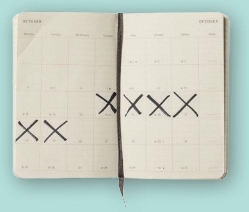

Fertility Awareness

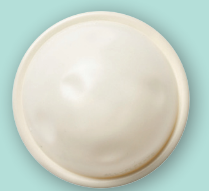

Diaphragm

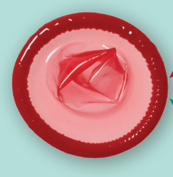

Condoms, for men or women

Needed for STD protection!

Use with any other method

For each of these methods to work, you or your partner have to use it every single time you have sex.

12-24 in 100 women, depending on method

FYI, without birth control, over 90 in 100 young women get pregnant in a year.

BEDSIDER
Bedsider.org

Bixby Center for Global Reproductive Health

CC BY NC ND

This work by the UCSF School of Medicine Bixby Center and Bedsider is licensed as a Creative Commons Attribution - NonCommercial - NoDeriv 3.0 Unported License.

Contraceptive Counseling Pocket Guide

Most Effective

	Implant	IUD (hormonal)	IUD (copper)	Sterilization
			Spermicide via inflammation	Interrupt vas deferens/tubes
	Irregular bleeding, ↓ flow & pain<br>Insertion risks (*e.g.*, infection, bruising)	Irregular bleeding, ↓ flow & pain<br>No ↑ risk of STIs<br>↓ risk of uterine cancer	Heavy/painful menses<br>No ↑ risk of STIs<br>Contraindicated with current PID/STI	Permanent<br>Surgical (female) or outpatient (male) procedure

Very Effective

	Pill (combined), Patch, Ring	Pill (progestin-only)	Shot
		= Plan B & Ella<br>& may inhibit ovulation	
	Predictable cycle control, ↓ flow & pain, extended cycle options<br>↓ acne<br>↓ risk of ovarian and uterine cancers<br>↑ thromboembolism risk with risk factors (*e.g.*, smoking, uncontrolled hypertension, migraine w/aura)	Requires consistent use at same time each day<br>Irregular bleeding, ↓ flow & pain<br>Only contraindication is current breast cancer	Irregular bleeding, ↓ flow & pain<br>Weight gain<br>Reversible ↓ bone mineral density<br>Delayed return of fertility

Effective

	Withdrawal	Fertility Awareness	Diaphragm	Condoms
	Removal of penis from vagina before ejaculation	Abstinence from end of menses to 3rd day of elevated basal body temp	Barrier<br>Used with spermicide	Barrier (internal or external)<br>Used with any other method
	No protection against STIs<br>Failure due to pre-ejaculate sperm	Requires regular, predictable cycles	Reusable<br>Insert up to 6 hours before intercourse, leave in for at least 8 hours after	Protection against STIs<br>Requires negotiation with partner

Key

- Common features for consideration (not an exhaustive list)
- Can be used for emergency contraception
- Requires office visit for insertion & removal
- Inhibits ovulation
- Thickens cervical mucus & thins uterine lining
- Decreases tubal motility

Counseling Best Practices

- Establish rapport
- Elicit patient's reproductive goals
- Explore patient's preferences for features of contraceptive methods; efficacy is not always the most important feature
- Clarify the meaning of numerical figures about risk and efficacy
- Share information about risks & side effects iteratively
- Anticipate barriers to consistent & correct use
- Address concerns respectfully
- Arrive at a shared decision
- Consider screening for reproductive coercion

REMEMBER: There is no single best method!

Clinician Resource: WHO Medical Eligibility Criteria Wheel (https://srhr.org/mecwheel/)

Created by Irene Tang and Devon Rupley, MD for the Scholarly Projects Program (2022).

Columbia | Vagelos College of Physicians and Surgeons
