# Supplementary material for: An Interactive Curriculum to Teach Person-Centered Contraceptive Counseling
Source: MedEdPORTAL. 2023 Dec 19;19:11368. doi: 10.15766/mep_2374-8265.11368 (PMC10728363; doi:10.15766/mep_2374-8265.11368)
Supplement: Supplementary file 1 — Contraceptive Options Chart and Pocket Guide.pdfPerson-Centered Contraceptive Counseling Module folderCase Development Tool.docxFacilitator Information and SP Training.docxFormative Session Checklist.docxPre- and Postsurveys.docx [file mep_2374-8265.11368-s001.zip › B. Person-Centered Contraceptive Counseling Module.zip!/B. Person-Centered Contraceptive Counseling Module/B. Person-Centered Contraceptive Counseling Module/scormcontent/index.html]

Person-Centered Contraceptive Counseling
